# Injuries of the Head

**Published:** 1878-06

**Authors:** 


INJURIES OF THE HEAD.—Every practical surgeon knows the difficulty sometimes attending the diagnosis of injuries about the head, especially when the patient has been drinking before the receipt of the injury. An unfortunate answer to the question, "Is he drunk or dying?" has frequently brought upon our police surgeons no measured censure. In this connection it is interesting to note the opinion of so eminent a surgeon as Mr. Erichsen, who says there is not one absolute method of diagnosis, except by waiting to see the result. "It is impossible," he says, "for any physician or surgeon to discover off-hand and say what the exact cause of the symptoms of coma or compression of the brain may be. This diagnostic point cannot be established by any practitioner, however skilled, or however experienced, unless you give him time." In order to relieve the surgeon of responsibility, Mr. Erichsen suggests the establishment of wards for the reception of such cases, that they may neither disturb the other patients, nor die of neglect.—*The Lancet*, 1878.

